# Influence of Yeast Products on Modulating Metabolism and Immunity in Cattle and Swine

**DOI:** 10.3390/ani11020371

**Published:** 2021-02-02

**Authors:** Nicole C. Burdick Sanchez, Paul R. Broadway, Jeffery A. Carroll

**Affiliations:** Livestock Issues Research Unit, ARS, USDA, Lubbock, TX 79403, USA; rand.broadway@usda.gov (P.R.B.); jeff.carroll@usda.gov (J.A.C.)

**Keywords:** immune response, livestock, metabolism, yeast

## Abstract

**Simple Summary:**

Dietary supplementation has been used in order to improve animal growth and health, and reduce the risk of disease for many years. Yeast and yeast products are a group of supplements that have broad applications in livestock production. These benefits include improving milk production, weight gain, and immunity. Recent studies suggest that yeast can have impacts beyond growth and health and may impact metabolism. Available energy is important for immune activation, and therefore any change in metabolism and energy availability may affect immune responses. This paper explores the effects of yeast on energy metabolism and how these changes may influence immune responses in cattle and swine.

**Abstract:**

Nutritional supplementation has been used by livestock producers for many years in order to increase animal performance, improve animal health, and reduce negative effects associated with enteric and/or respiratory pathogens. Supplements such as yeast and yeast-based products have broad applications across many livestock production systems, including poultry, aquaculture, cattle, and swine and have been shown to benefit animal production at various stages. These benefits include improvement in milk production, weight gain and feed conversion, as well as immune function. Initial research into the mode of action for these effects has focused on stimulation of the immune system by the β-glucan fractions of yeast. However, emerging studies have revealed that some of the beneficial effects of yeast products may stem from altering metabolism, including the availability of glucose and fatty acids. These changes in metabolism, and potentially energy availability, may partially explain differences in immune function observed in yeast-supplemented livestock, as the energy demands of an activated immune system are extremely high. Thus, this paper explores the influence of yeast products on metabolism in cattle and swine, and how changes in metabolism and energy availability may contribute to improvements in immune function in supplemented animals.

## 1. Introduction

Livestock production is currently in a period of constant flux. The ever-increasing demand for livestock raised without synthetic compounds and pharmaceuticals, such as growth promoters and antibiotics, has driven producers to seek more innovative and ‘natural’ approaches to production. These approaches include utilizing feed supplements that provide growth benefits as well as health benefits, and often include or contain yeast or yeast by-products. Consequently, there has been an influx of supplements in the feed market that claim to enhance performance or alter health. Indeed, there are many yeast products that have been demonstrated to improve growth, feed efficiency, milk production, and immune responses [1]. However, other yeast products, under certain conditions, yield limited or negative effects on livestock production [2,3].

Additionally, there is data to suggest yeast supplementation affects more than immunity and growth, with recent evidence implicating a role for yeast in altering metabolic responses [4,5]. Metabolism can often be overlooked with regard to its role on immune function. The immune system requires a substantial amount of energy once activated in order for the body to successfully defend itself against an invading pathogen, which includes production of antibodies, complement and acute phase proteins (APP). Estimates suggest this energy demand could be increased as much as 10–30%, with some estimates even higher at 55% with respect to required metabolizable energy needed by the body to activate the immune system [6,7,8]. This energetic requirement is supported by studies that have reported the immune systems of cattle and swine use approximately 1 kg of glucose within a 12-hour period when activated [9,10].

With the increasing consumer demand to eliminate synthetic pharmacological compounds from livestock production and ensuing legislative pressure [11], yeast products may provide benefits to livestock producers that poise them to serve as an alternative to antibiotics and other synthetic supplements given the increased focus on antibiotic resistance and ineffectiveness. Through an understanding of how these products elicit their effects, we can provide better information to producers in order to target areas of livestock production where these products may have their greatest benefit. This review provides an overview of yeast products available to producers, their suspected mode of action, and how these products have been found to affect immunity and metabolism in cattle and swine.

## 2. Yeast Products

There are a wide variety of yeast and yeast by-product supplements available for use in livestock production systems. In fact, yeast are the most common probiotic supplement fed to dairy cows [12,13]. Yeast supplements include live yeast, yeast cell wall (YCW), purified cell wall components such as monooligosaccharide (MOS) and β-glucans, as well as yeast fermentation or culture products that are by-products produced from the fermentation of yeast. These products differ in appearance, composition of biologically active components, and production system application. For example, some of these products may be used specifically to increase milk production in the dairy cattle industry [14], while other products are focused more on improving growth or health [1]. Additionally, the conditions (e.g., temperature, nutrient source, incubation period) under which the yeast are propagated or fermented, as well as the serotype or strain of yeast used, can greatly influence the end product and the subsequent effects when fed to livestock. Thus, it is important to understand the differences in available products when selecting a yeast supplement.

There are several ways in which yeast products elicit their effects, including enhancing intestinal barrier function, altering bacterial populations within the gastrointestinal (GI) tract, and through providing beneficial substrates for host bacteria. Altering GI tract permeability by enhancing nutrient absorption, while limiting pathogenic bacteria translocation and release of toxins systemically, can greatly benefit the host through reducing inflammation. This is often a result of altering mucin production, tight junctions between epithelial cells, and crypt and villi characteristics within the GI tract. For example, yeast-supplemented pigs were found to have reduced intestinal mucus thickness compared to control pigs, which may improve absorption of nutrients [15,16]. Additionally, increases in villus height and crypt depth, both measures of intestinal health, have been found in weaned pigs supplemented with yeast [16]. In weaned dairy calves, YCW supplementation improved the development of the GI tract [17]. The combination of yeast (*Saccharomyces cerevisiae*) and the probiotic *Bacillus licheniformis* reduced the permeability of the small intestine in pigs challenged with *Escherichia coli* K88, demonstrating improved intestinal integrity and barrier function [18]. The reduction in systemic and/or sub-clinical inflammation may reduce the energy demand by the immune system and may improve performance.

Yeast supplementation can also improve rumen stability in cattle through increasing rumen pH [19]. Pinloche, et al. [20] observed increases in the volatile fatty acids (VFA) propionate and butyrate yet observed decreases in lactate and ammonia in lactating dairy cows supplemented with yeast. This is beneficial as lactate, produced as a result of feeding cereal grains, can decrease pH resulting in acidosis, a significant health problem in ruminants [21]. Through a reduction in lactate, yeast supplementation acts to increase the pH of the rumen and allows for a ruminal environment more conducive to the growth of beneficial bacteria [22]. For example, supplementation of yeast to dairy calves increase the population of *Ruminococcus albus*, which degrade cellulose [23]. Additionally, yeast is able to reduce concentrations of pathogenic bacteria within the GI tract [24,25]. In cattle, it is common to feed high-grain diets in order to improve performance but it comes at the expense of an increase in incidence of acidosis which is associated with decreases in dry matter intake (DMI) and rumen pH, and increases in inflammation and gut barrier function disruption [26]. Yeast was found to change gene expression of immune-related genes in the rumen both prior to and following calving in cows, which may be a means of regulating inflammation and gut barrier integrity during this transition period [27]. Further, yeast have been found to not only increase cellulolytic bacteria that break down indigestible fiber, but yeast also scavenge oxygen within the rumen, potentially playing another role in stabilizing pH and promoting digestion of fiber-based feedstuffs [13,20,28,29]. Yeast can also play a role in improving the GI development in cattle and swine, particularly in the pre-weaning and early post-weaning period [15,30], which may provide benefits to the animal throughout the subsequent growth period.

While yeast can support beneficial bacteria, there is also evidence demonstrating yeast can target pathogenic bacteria within the GI tract [13]. Posadas et al. [31] observed that yeast and YCW products can directly bind to pathogenic bacteria, including *Salmonella, Escherichia coli, Clostridia, Listeria,* and *Fusobacteria*, with the response being strain-specific. Additionally, the proliferation of yeast may provide nutrients (e.g., organic acids and growth factors) to beneficial bacteria within the GI tract, thus acting as a prebiotic. Yeast fermentation or culture products aim to harness this potential by supplying the GI tract with nutrients that can be used to promote the growth of beneficial bacteria. Yeast prebiotics, such as purified YCW products, are also used to promote the growth of beneficial bacteria. Yeast cell wall is rich in MOS and β-glucans, which act in similar ways as live yeast to prevent binding of bacteria to the GI tract [1]. Additionally, yeast based prebiotic products can also improve the pH balance and overall microbial health of the GI tract.

While there are different mechanisms of action of yeast, a general theme appears to be improvement in gut health, including improved gut integrity and maintenance of a healthy microbiome. It is possible that, through these actions, yeast can reduce inflammation, leading to increased nutrient absorption, reduced low-grade inflammation, and thus improved overall animal performance. While there are benefits of feeding yeast products within the GI tract, there have been numerous reports of systemic immune system benefits. However, as noted below, these effects are dependent upon the product and dose administered, the stage of animal production in which supplementation is initiated, and the overall health status of animal. As such, some yeast product supplementation may also yield limited or negative effects [32,33].

## 3. Immunity and Metabolism

The immune system can be separated into two broad categories, the innate and adaptive immune system. The innate immune response is relatively non-specific, and includes cellular barriers, white blood cell (WBC) populations (i.e., macrophages and neutrophils), and secreted factors such as cytokines and compliment that work together in order to kill or neutralize pathogens upon detection. If the innate immune system is unable to control the infection, the adaptive immune system is activated, resulting in the stimulation of T and B lymphocytes and subsequent pathogen-specific cellular killing and/or antibody production. Under normal circumstances, the immune system is a coordinated response aimed at dispatching invading pathogens, thus allowing the host to return to normal maintenance and growth behaviors. However, this response can be altered during periods of intermittent or chronic stress such as weaning, thermal stress or transportation events.

Upon activation of the immune system, changes occur within the body to redirect energy utilization towards the immune system and to reduce energy expenditure that may negatively impact the activated immune system [34]. Energy conserving activities include a decrease in appetite and an associated decrease in digestion, altered behavior such as malaise and depression, and other clinical sickness behaviors [35,36]. These are necessary steps in order to conserve energy, particularly glucose, which the immune system needs [34]. The dependence of the immune system on metabolic processes and energy availability is often overlooked but is beginning to be studied in greater detail. Studies in cattle and swine have found that the immune system utilizes approximately 1 kg of glucose within the first 12 h of an experimental immune challenge with lipopolysaccharide (LPS) [9,37]. The relative conservation of this amount of glucose across species is interesting due to the differences in glucose production between cattle and swine, and highlights the importance of glucose as a source of energy to the immune system. Additionally, an immune challenge can result in increases in gluconeogenesis up to 150–200% [8]. There are various estimates of the energy required by an activated immune system, and the requirements of the innate immune system differ from those of the adaptive immune system. For example, it is reported that the acute phase response requires more energy than the production of antibodies [38]. Humans have been reported to need 7–15% more energy consumption for every 1 °C increase in body temperature [7], while humans in a state of septicemia utilize 30–60% more energy [39]. Huntley, et al. [40] reported that pigs experimentally challenged with LPS used 24% more metabolizable energy than non-challenged pigs. Unfortunately, it is difficult to fully measure the amount of energy utilized by an activated immune response, but these estimates make it clear that the energetic requirement of the immune system is biologically significant to the host animal. Thus, any increase in available energy may provide a tremendous benefit to an animal during an immunological challenge, potentially altering the timeline of illness/disease resolution and recovery.

Interestingly, there is data to support that the immune system can also utilize other forms of energy rather than glucose, such as fatty acids [41]. In fact, changes in the cellular metabolism of immune cells, including substrate availability, can influence their phenotype, resulting in a pro- or anti-inflammatory phenotype [41,42,43]. For example, classically activated macrophages (M1) utilize glucose and glycolysis and are pro-inflammatory, while alternatively activated macrophages (M2) are anti-inflammatory in nature and utilize fatty acid oxidation to drive the Krebs cycle for energy rather than glycolysis [44]. Additionally, Th17 cells, which are inflammatory in nature, rely on glycolysis while T-regulatory (T-reg) cells, which are mainly anti-inflammatory, rely on fatty acid oxidation [45]. There are also differences in metabolism between activated T lymphocytes and memory T lymphocytes, where the former utilize oxidative phosphorylation and glycolysis and the later utilize β-oxidation [46]. Thus, available energy sources can truly shape the immune response, and may direct the subsequent recovery period. These discoveries have resulted in increased research of cellular metabolism in the focus area termed immunometabolism.

Yeast and yeast-based products have been demonstrated to alter immune function, including altering WBC and cytokine concentrations, although responses appear to be dependent on the type of product used, stage of animal production, and overall health of the animal. Further, more recent evidence suggests a role of yeast products in altering metabolism, a response that appears to be more consistent regardless of the type of yeast supplement utilized. The following sections will discuss the impact of yeast supplementation on the immune response, as well as recently identified metabolic changes observed in response to supplementation of yeast products.

### 3.1. Modulation of Immune Function by Yeast

Supplementation of cattle and swine with yeast has produced varying responses by the immune system (Table 1). As discussed above, the immune system can be divided into the innate response, consisting of the initial, relatively non-specific response to a pathogen, and the adaptive response, which is a pathogen-specific response. Both of these immune responses have been modulated by yeast supplementation.

Changes in WBC populations can be indicative of an infection. Thus, any change observed in WBC populations may signify an improvement or worsening of the condition. In cattle and swine, lymphocytes are the most populous WBC subtype, followed by neutrophils. In response to a vaccine challenge, steers had a significant decrease in circulating neutrophils and lymphocytes, but this response was prevented in steers supplemented with a hydrolyzed yeast [47]. In contrast, beef steers supplemented with a *Saccharomyces cerevisiae* fermentation product (SCFP) prior to challenge with LPS were found to have greater concentrations of platelets and WBC populations, yet had reduced pro-inflammatory cytokine concentrations following the immune challenge [48]. The authors suggested that the greater WBC populations prior to the LPS challenge served to prime the immune system, preparing it for the challenge, and ultimately resulted in the decreased cytokine concentrations observed post-challenge. Yet, supplementation with a combination live yeast and YCW product resulted in no change in WBC concentrations prior to or following a viral-bacteria respiratory disease challenge in heifers [62]. The varied responses observed in cattle may be attributed to the type of product supplemented, the general health of the cattle, or the stage of cattle production. Additionally, some of the differences observed may be due to sexual dimorphism (steers versus heifer in the aforementioned studies), as immune responses have been demonstrated to differ as a result of sex [63].

In sows, supplementation with SCFP reduced concentrations of total WBC as well as neutrophils [52]. Similarly, WBC concentrations, including neutrophil and lymphocyte subsets and the neutrophil to lymphocyte ratio, were reduced in pigs fed two different doses of YCW prior to challenge with *Salmonella typhimurium* [53]. Decreases in the neutrophil to lymphocyte ratio can be indicative of reduced inflammation due to the inflammatory nature of neutrophils within tissues. In contrast, pigs supplemented with yeast and subsequently challenged with E. coli were found to have no differences in total WBC populations following the challenge [61]. Yet, weaned pigs supplemented with yeast had increased numbers of WBC but reduced pro-inflammatory cytokine concentrations relative to an LPS challenge [54]. Thus, similar to cattle, there appear to be varying responses in WBC populations relative to yeast supplementation in swine. Studies have also analyzed changes in immune cell subtypes. For example, weaned pigs supplemented with yeast culture had reduced CD4+ cells, or T-helper cells [55]. However, weaned pigs supplemented with dietary β-glucan isolated from yeast were found to have increased CD4+ cells [56]. These studies demonstrate differences in specific WBC populations are also influenced by the yeast supplement and the challenge model used.

While changes in WBC populations can provide information about potential infections, another aspect of immunity is the ability of WBC to kill or neutralize pathogens. Indeed, changes in functional aspects of WBC have also been observed in response to yeast supplementation. Specifically, increased oxidative burst and phagocytosis were observed in neutrophils isolated from Holstein steers that had been supplemented with yeast [50,51]. Additionally, macrophages isolated from the lamina propria of weaned pigs supplemented with phosphorylated mannans (isolated from yeast) had greater phagocytic capacity compared to macrophages isolated from non-supplemented pigs [64]. However, no differences in neutrophil or macrophage function were observed in weaned pigs supplemented with β-glucan [65].

Activation of the immune system, including recruitment of WBC and stimulation of sickness behavior, are dependent upon the secretion of cytokines. Pro-inflammatory cytokines, such as tumor necrosis factor-α (TNF-α), interleukin-6 (IL-6), and interferon-γ (IFN-γ) stimulate fever, sickness behavior, APP production, and also stimulate production of other cytokines to support the immune response. In contrast, anti-inflammatory cytokines, such as IL-10 and IL-4, act to reduce inflammation and also activate the adaptive immune system. Thus, cytokine concentrations can be an important indicator of systemic inflammation and immune system activation. Supplementation of beef heifers with two different YCW products resulted in a decrease in both magnitude and duration of serum concentrations of IL-6 following an LPS challenge compared to non-supplemented heifers [2]. This cytokine response was complemented by reduced rectal temperatures that were observed in the study, suggesting an overall reduction in the inflammatory response. In contrast, supplementation of weaned pigs with SCFP increased serum concentrations of the pro-inflammatory cytokines TNF-α and IL-6, yet cytokines were elevated for a similar duration of time compared to non-supplemented pigs [57]. Supplementation of β-glucan to weaned pigs prior to an LPS challenge resulted in reduced concentrations of IL-6 and TNF-α, but greater concentrations IL-10 within the acute (3 to 6 h) period following LPS administration [59]. This cytokine response is suggestive of a reduction in the pro-inflammatory response and an increase in the anti-inflammatory response which the authors proposed may support the improved pig performance observed in the study. Similarly, weaned pigs supplemented with MOS and subjected to a porcine reproductive and respiratory syndrome virus challenge were found to have reduced concentrations of TNF-α yet greater IL-10 concentrations post-infection, and also had greater concentrations of WBC in the early period post-infection [58]. Perhaps the greater concentrations of WBC observed early post-infection reduced the pro-inflammatory cytokine response in this particular study. Thus, similar to observed changes in WBC populations, there is variation in serum cytokine concentrations based on product and challenge model, and differences in both cytokine magnitude and duration are likely due to these factors. Additionally, while cytokines are important for the immune response, overstimulation of pro-inflammatory cytokines for an extended period of time can result in a hyperinflammatory state that can be detrimental to the health and recovery of the animal.

Acute phase proteins, produced in abundance between 12 to 48 h following activation of the immune system, act to support the immune response during an infection. The primary APP measured in cattle and swine are haptoglobin, serum amyloid A, fibrinogen and C-reactive protein, and are produced in the liver as a result of cytokine stimulation. The APP act in various ways to support the immune response, including binding pathogens, activating complement, and binding cellular debris [66]. Additionally, APP may play a role in supporting the immune system through altering lipid metabolism within the liver [67]. Concentrations of haptoglobin, serum amyloid-A, LPS-binding protein and C-reactive protein were reduced in beef steers supplemented with YCW compared to non-supplemented steers following 45 days of supplementation [49]. Yet, supplementation with a combined live yeast and YCW product resulted in no difference in haptoglobin concentrations in beef heifers following a combined viral-bacterial respiratory disease challenge [62]. However, the authors suggested that peak haptoglobin values may not have been realized due to the sample collection interval. In contrast, beef steers supplemented with SCFP had greater serum concentrations of fibrinogen compared to non-supplemented steers following an LPS challenge [48]. Fomenky et al. [51] also observed greater concentrations of serum amyloid A and C-reactive protein in Holstein steers supplemented with yeast during the weaning period. The increase in APP in the two later studies suggests this may be due to stimulation of the immune response or stress response due to the immune challenge or the stress of weaning. Many APP, including serum amyloid A and C-reactive protein, can inhibit immune responses in an effort to reduce inflammation [67]. This is contrasted to the decreased APP concentrations in the study by Lei et al. [49], as no immune challenge or stressor was present, and therefore supplementation with YCW reduced general inflammation including APP. Thus, the differences in APP responses are likely indicative of the differential biological needs of the animals under different physiological states and immune challenges.

One common method for measuring the status of adaptive immunity is through the measurement of antibodies, either produced in response to infection or vaccination, or those passively delivered to neonatal animals via their dam’s colostrum shortly after birth. Antibodies bind to pathogens and infected cells, particularly bacterial pathogens, targeting them for destruction through complement binding or phagocytosis by WBC. In pigs supplemented with β-glucan, an increased antibody response was observed following immune stimulation with ovalbumin, suggesting a stronger adaptive immune response [60]. Live yeast increased serum immunoglobulin A (IgA) concentrations in weaned pigs [68,69]. Piglets were also observed to have greater plasma IgG concentrations 24 h after birth when sows were supplemented with live yeast [70]. However, no difference in serum IgG concentrations were observed in the colostrum or milk of sows supplemented with SCFP throughout gestation or in the serum of their piglets measured 1 and 17 days after birth compared to non-supplemented sows and piglets [52]. It is possible that yeast fermentation products, which contain limited yeast, are not able to stimulate the adaptive immune response in similar ways compared to live yeast or purified yeast β-glucan, but this area requires additional study. Supplementing gestating cows with yeast culture resulted in greater serum concentrations of IgG two days post-calving in both the cows and their calves compared to non-supplemented cows and their calves [71]. Similarly, supplementation of dairy cows with yeast culture 90 days prior to and following parturition resulted in greater serum concentrations of IgG, IgM, and IgA in cows and their calves at 0 and 24 h after birth compared to non-supplemented cows and calves [72]. Thus, it appears that yeast supplementation may provide a benefit to an animal through increased production of antibodies, either due to immune challenge or through increased antibody concentrations within the colostrum.

While the above paragraphs detail the specific immune responses observed in the presence of yeast and yeast-based products, other general effects of yeast products on immunity have been noted. These include changes in diarrhea incidence, as well as changes in morbidity and mortality in cattle and swine. For example, live yeast and yeast culture have been observed to decrease incidence of diarrhea, reduce antibiotic use, and improve survival in dairy calves [73,74,75]. Changes in disease incidence, outside from notable or recorded changes in immune parameters, may be viable indicators of changes in immunity in response to yeast product supplementation.

It is also important to note that there is no single response that is indicative of a “good” immune response, as both changes in magnitude and duration may suggest improvement or deterioration. Further, there has yet to be identified one single immune parameter that is able to classify an animal as healthy or sick. In fact, while immune factors above were discussed in specific categories (e.g., cytokines, WBC, antibodies), it is actually the complete immune response, or the integration of these factors, that is needed to fully understand and quantify the immune response. The best indicator of an improved immune response is a decrease in the time it takes for an animal to recover and return to normal maintenance behaviors. This implicates a role of the immune response, but also the metabolic response and energy availability, as a significant reduction in available energy and nutrients will prolong the immune response and increase the time needed for an animal to recover performance losses.

### 3.2. Yeast Effects on Metabolism and Potential Modes of Action

While substantial variability in the immune response has been observed when cattle and swine were supplemented with yeast products, there appears to be a more consistent metabolic response (Table 2). Yeast cell wall supplementation of weaned beef steers altered the metabolic response following LPS challenge, where greater glucose and insulin responses were observed yet concentrations of non-esterified fatty acids (NEFA) were reduced [76]. Greater glucose responses were also observed in steers supplemented with SCFP subsequent to an LPS challenge [48]. These two studies suggest that supplementation of yeast products may improve energy availability during an immune challenge, which may be beneficial to allow for a more rapid resolution. Similarly, greater glucose concentrations were observed in Holstein calves with failure of passive transfer following supplementation with yeast [73]. Additionally, lactating dairy cows supplemented with live yeast or yeast culture had greater serum glucose and reduced blood urea nitrogen compared to control cows [5,77]. In contrast, beef heifers supplemented with a combination live yeast and YCW product had similar glucose concentrations compared to non-supplemented heifers, yet had reduced concentrations of urea nitrogen in response to a respiratory disease challenge [62].

It is interesting to observe the relatively consistent effect of yeast supplements on glucose concentrations. This contrasts with some of the immune parameters discussed above, where different responses in circulating WBC and cytokines were observed across yeast products and animal species. There are several possible explanations for the increase in glucose concentrations. Doležal et al. [5] suggested that supplementation with yeast improved fiber digestion, thus increasing blood glucose concentrations and resulting in improved energy status. In support of this, active dry yeast may play a role in maturing the microbiota of the rumen by increasing the number of cellulolytic bacteria [28]. An increase in the number of lactate utilizing and fibrolytic bacteria were observed in cows supplemented with live yeast [20]. This is proposed as one of the modes of action of yeast and may result in a more stabilized pH as noted earlier. Increases in fibrolytic and cellulolytic bacteria promote digestion of fiber-based feedstuffs which not only stabilizes rumen pH, but also increases the utilization of ingested fibrous feedstuffs. Additionally, yeast products, such as SCFP, have been demonstrated to increase rumen propionate concentrations, which may play a role in increasing serum glucose concentrations in cattle [79]. Greater total VFA and acetate were observed in dairy cows supplemented with live yeast, thus providing increases in substrate availability for microbial glucose production [80]. Additionally, glucose was greater and NEFA lower at peak lactation compared to non-supplemented cows, suggesting a role for yeast in alleviating the burden of a negative energy balance. Regardless, it appears that yeast play a role in increase glucose concentrations, which may provide additional energy that could be utilized during an immune response and potentially accelerate recovery from an infection.

There was a tendency for concentrations of plasma urea nitrogen to be reduced in sows fed SCFP in gestation and lactation, suggesting this product may reduce protein catabolism [52]. In addition, concentrations of NEFA, typically viewed as an indicator of fat catabolism, were reduced in weaned pigs supplemented with YCW [53]. Increases in fat and protein catabolism may result in a decrease in carcass tissues, ultimately affecting hot carcass weight and profitability. However, changes in these parameters can also be influenced by feeding behaviors and ruminal digestion. Regardless, the reduced urea nitrogen concentrations reported in the aforementioned studies support improved protein utilization and/or reduced protein degradation in cattle supplemented with yeast. However, it is unclear by what mechanism yeast supplementation is improving protein utilization or reducing protein degradation, an area which would benefit from additional research. Reduced concentrations of urea nitrogen and NEFA compliment the greater glucose concentrations observed in yeast-supplemented animals, and suggests that the greater glucose reduces the utilization and catabolism of adipose and protein. Additionally, reducing the need to utilize protein and fatty acids for energy via gluconeogenesis is important as both fatty acids and amino acids can be used by the immune system for the production of immune mediators.

While not the focus of the current review it is worth mentioning the varying responses of yeast products on performance, specifically dry matter intake (DMI), as this response may affect other metabolic responses to yeast supplementation [29,77]. The increase in feed intake observed in many studies through yeast supplementation may be a result of a decrease in lactic acid, preventing a decrease in rumen pH and subsequently allowing for the growth of fibrolytic and cellulolytic bacteria which improve digestion [12,29], as discussed above. The increase in DMI, therefore, may be one of the driving factors associated with the increase in VFA and glucose production. Additionally, dairy calves were observed to have greater rumen propionate percentage and ammonia nitrogen when supplemented with yeast, indicative of increased available energy and providing support for the increased glucose and insulin concentrations observed [81]. However, Dehghan-Banadaky et al. [77] reported increased glucose concentrations in yeast-supplemented lactating cows in the absence of any difference in DMI compared to non-supplemented cows. Yet, this study also reported increased neutral detergent fiber which supports the increase in glucose concentrations. Further, increased energy availability may also decrease NEFA and β-hydroxybutyric acid (βHBA) through a reduction in lipid mobilization [80]. This is important as a rise in these variables, such as during the early post-partum and lactation period, has been associated with increases in disease incidence [26,82]. Additionally, there is data to suggest that addition of yeast can influence immunity and metabolism within the rumen. Petri, et al. [83] reported increases in the expression of cell metabolism and nutrient transport genes within the rumen epithelium. The authors discussed that this is supported by increases in DMI and is suggestive of an increase in removal of rumen SCFA.

Therefore, it appears that yeast supplementation may provide a substantial benefit to an animal during an immune challenge through improving available energy via increasing glucose, thus reducing the catabolism of adipose and protein. Through these mechanisms, yeast may be able to support the immune system and improve recovery from an immunological insult. Additional research is necessary in order to more fully understand the mechanism by which yeast products increase serum concentrations of glucose and decrease fatty acid and urea nitrogen concentrations.

## 4. Conclusions

In conclusion, while there appears to be convincing evidence that yeast products can improve performance and modulate immune function, the mechanisms behind these effects are still under investigation. More recent evidence suggests a role of yeast products in altering metabolism, particularly with regard to energy availability. These metabolic changes may improve immune responses in cattle and swine supplemented with yeast. This area is supported by the ongoing research that is working to understand the energy demand of the immune system both at rest and following activation.

There is still information that is lacking on the application of yeast products. First, the length of time supplementation is needed prior to exposure to a pathogen to prevent subsequent infection or reduce the severity of infection is not clearly known and appears to vary from a few days to up to three weeks. Thus, feedlot producers aimed at supplementing cattle to reduce the incidence of sickness during the receiving period may be at a disadvantage if cattle are not fed prior to feedlot arrival. A second area that is unclear is whether the benefits of yeast product supplementation are maintained following cessation of supplementation, and if not, how quickly the advantage dissipates. Lastly, information on the application of yeast products across various stages of production is lacking. Therefore, there is a need for additional research to better understand and identify application methods that provide the greatest benefit to the particular livestock production stage.

As we gather a better understanding of the mechanisms by which yeast products improve performance, immunity, and metabolism, these products can be better utilized, both within and across production systems, to improve livestock production and health. Additionally, it is possible that yeast products, as well as other pre- and probiotic supplements, may play a role in maintaining livestock productivity during a rapidly changing environment of antimicrobial stewardship and reduction. Continued research in the livestock area is necessary in order to continue to provide reliable options to livestock producers.

## Figures and Tables

**Table 1:** Effects of yeast and yeast-based product supplementation on the immune response in cattle and swine.

Species	Yeast Product	Effect on Immune Function	Reference
Beef cattle	Hydrolyzed yeast	Prevented decrease in WBC ^1^	[47]
Beef cattle	SCFP ^2^	Increased WBC and platelets; decreased pro-inflammatory cytokines; decreased fibrinogen	[48]
Beef cattle	YCW ^3^	Reduced IL-6 concentrations	[2]
Beef cattle	YCW	Decreased acute phase proteins	[49]
Dairy calves	Yeast	Increased neutrophil function	[50,51]
Sows	SCFP	Reduced leukocyte concentrations	[52]
Weaned pigs	YCW	Reduced leukocyte concentrations	[53]
Weaned pigs	Yeast	Increased WBC; reduced pro-inflammatory cytokine concentrations	[54]
Weaned pigs	Yeast culture	Decreased IFN-γ concentrations and CD4+ T cells	[55]
Weaned pigs	β-glucan	Increased CD4+ T cells	[56]
Weaned pigs	SCFP	Increased pro-inflammatory cytokine concentrations	[57]
Weaned pigs	MOS ^4^	Greater WBC; reduced cytokine concentrations	[58]
Weaned pigs	β-glucan	Greater cytokine concentrations	[59]
Weaned pigs	β-glucan	Reduced TNF-α and IL-6; Increased IL-10	[60]
Weaned pigs	Yeast	No change in leukocyte populations	[61]

^1^ WBC: white blood cells; ^2^ SCFP: *Saccharomyces cerevisiae* fermentation product; ^3^ YCW: Yeast cell wall; ^4^ MOS: monooligosaccharide.

**Table 2:** Effects of yeast and yeast-based product supplementation on metabolic parameters in cattle and swine.

Species	Yeast Product	Metabolic Parameter	Reference
Beef calves	YCW ^1^	Greater glucose and insulin; reduced NEFA ^2^	[76]
Beef calves	SCFP ^3^	Greater glucose	[48]
Beef calves	Live yeast and YCW	Similar glucose; reduced urea nitrogen	[62]
Dairy calves	Yeast	Greater glucose	[73]
Dairy cows	Live yeast or Yeast culture	Greater glucose; reduced urea nitrogen	[5,77]
Dairy cows	Live yeast	No effect on glucose	[78]
Weaned pigs	YCW	Reduced NEFA concentrations	[53]
Sows	SCFP	Tendency for reduced urea nitrogen	[52]

^1^ YCW: yeast cell wall; ^2^ NEFA: non-esterified fatty acid; ^3^ SCFP: *Saccharomyces cerevisiae* fermentation product.

## Data Availability

Not applicable.
